# Supplementary material for: Targeting mTOR Pathway in PTEN Deleted Newly Isolated Chordoma Cell Line
Source: J Pers Med. 2023 Feb 27;13(3):425. doi: 10.3390/jpm13030425 (PMC10056194; doi:10.3390/jpm13030425)
Supplement: Supplementary file 1 [file jpm-13-00425-s001.zip › jpm-2231666-supplementary.pdf]

Table S1. Primary antibodies used in the study.

Primary Antibody	Company	Catalog #	Host	Clone	Dilution
Brachyury	Santa Cruz Biotechnology	Sc-374321	Mouse	A-4	1:200
Cytokeratin (CK-Pan)	Dako	M0821	Mouse	MNF116	1:200
EMA	Leica Biosystems	PA0035	Mouse	GP1.4	1:150
INI1-BAF47	Becton Dickinson	612110	Mouse	25/BAF47	1:200
Ki67	Dako	M7240	Mouse	MIB-1	1:100
pNDRG1 (Thr346)	Cell Signaling	5482	Rabbit	D98G11	1:100
pS6 (Ser235/236)	Cell Signaling	4857	Rabbit	91B2	1:100
PTEN	Dako	M3627	Mouse	6H2.1	1:100
